# Getting psyched up: Developing the field of Sport and Exercise Psychiatry in South Africa

**DOI:** 10.17159/2078-516X/2023/v35i1a16352

**Published:** 2023-09-14

**Authors:** JW Burger, B Mafuze

**Affiliations:** 1 HIV Mental Health Research Unit, Division of Neuropsychiatry, Department of Psychiatry and Mental Health, University of Cape Town, South Africa; 2 Department of Psychiatry and Mental Health, Nelson R Mandela School of Medicine, University of KwaZulu-Natal, South Africa

**Keywords:** mental disorders, mental health, athletes, sports psychiatry

## Abstract

Elite athletes and their entourage are exposed to intense stressors and commonly experience mental health symptoms and disorders. While there is limited literature on prevalence rates in a South African setting, initial data show that both current and retired athletes may experience these symptoms, but with low treatment rates. There is a need to improve mental health literacy and mental health care in athletes. Athletes may benefit from systematic mental health surveillance and additional screening during high-risk periods, such as injury or retirement. This commentary brings athlete mental health into focus and advocates for the inclusion of sport and exercise psychiatry into the athlete entourage and broader mental health team, in order to close the treatment gap. We provide seven recommendations for the initial way forward for sport and exercise psychiatry in South Africa.

South Africans love their sport. We tend to idolise our athletes as national heroes, viewing them as the individuals and teams who are capable, perhaps even expected, to bring together a nation. From travelling the world, to hearing fans chant their names on an international stage, athletes may seem to have it all. However, athletes are people first. They experience general personal stressors and the additional competitive and logistic pressures of being an elite athlete. There is increasing recognition that elite athletes experience mental health symptoms and may develop mental health disorders.^[1]^ This commentary aims to bring the mental health of the athlete into focus and provide recommendations for addressing challenges in a South African setting.

## Mental health symptoms and mental health disorders

Mental health symptoms and disorders occur in former and current elite athletes at similar rates to that of the general population.^[2]^ Mental health disorders are defined by the International Olympic Committee (IOC) consensus statement (2019) as conditions that meet the diagnostic criteria of the Diagnostic Statistical Manual of Mental Disorders (DSM) or the International Classification of Diseases (ICD) and cause clinically significant distress or impairment.^[1]^ Mental health symptoms are more common, referring to the wider spectrum of negative thoughts, feelings, and behaviours that do not necessarily meet criteria for a DSM or ICD disorder and may not necessarily cause clinically significant distress or impairments.^[1,3]^ Most of the research in athletes investigates mental health symptoms rather than mental health disorders.^[2]^

## The South African setting

Mental disorders have been a silent epidemic internationally, with low- and middle-income countries (LMIC) carrying a large but often neglected burden. Mental health disorders develop where a genetic predisposition meet environmental factors, such as trauma, stress, or substance misuse. Social determinants of mental health are key considerations in LMIC and South Africa, where social and health disparities are prominent. Research on prevalence rates of mental health symptoms and disorders in South African athletes is limited but seems to echo the trends of the international literature. South African populations have been included in international studies of retired^[4]^ and current professional rugby players^[5]^, with around 30% of them reporting mental health symptoms. South African para-athletes may be a group that requires specific attention, as 75% of current para-athletes at the National Championships reported high levels of psychological distress.^[6]^ The early data on mental health disorders in South African athletes show that one-third of a sample of current South African club rugby players had a diagnosable mood or anxiety disorder, with 10% having a current disorder.^[7]^

There are challenges with how best to link athletes with mental health disorders with the appropriate level of care, particularly within an LMIC setting. This echoes a broader challenge in the South African mental health landscape. Africa has a large treatment gap for mental health disorders, where there is a gap between the need for services and those who access treatment.^[8]^ These disparities stem from a lack of resources, training, facilities, and policy development. This treatment gap also shows itself in South African sports settings.^[7]^

## Mental health literacy

Stigma is a significant barrier to accessing help for mental health symptoms and disorders. While performance psychology has helped to destigmatise difficult emotions in the sporting world, we still have a long way to go in reducing the stigma of mental disorders, both in athletes and in the general population. We have seen several high-profile cases of successful elite athletes disclosing their struggles with mental health symptoms, including Michael Phelps, Serena Williams, and local stars, Lenize Potgieter and Sbu Nkosi. At times, though, the extent of their struggles is only identified after the fact, or when the athlete has died by suicide.

There is early evidence that mental health literacy can be improved through specific interventions, with better knowledge and more efficacious use of referral pathways.^[9]^ Research needs to explore interventions targeted to our local context to ensure that these are feasible and acceptable to our diverse athletes and coaching populations. These would aim to improve mental health literacy in South African sport, with upskilling to allow athletes and their entourage to identify signs and symptoms of mental disorders. With adequate mental health literacy, our athletes and coaches would be empowered to manage their mental health better and equipped with the knowledge of when and how to access help.^[10]^ Through engaging with the challenging themes of mental health and identity in a diverse African society, sports mental health can be an opportunity to open conversations across typical cultural and language divisions.

## Systematic mental health surveillance and high-risk periods

There is a large cost to not managing mental health disorders, both from a human and an economic perspective.^[11]^ Elite sport requires athletes to be healthy, both physically and mentally. The IOC has recently published recommendations for mental health surveillance, including standardised approaches to the recording of severity, symptom clusters, precipitants, and other pertinent factors.^[3]^ Ideally, screening and surveillance should be conducted systematically in sports systems, as well as in high-risk periods, such as after injury.^[3]^ Practitioners may decide to implement screening in their specific setting using the IOC Sport Mental Health Assessment Tool-1^[12]^ or where resources are limited, they may opt for the pragmatic use of tools already being performed, such as the symptom scale of the Sport Concussion Assessment Tool.^[7]^

For athletes, career transitions out of sport are often high-risk periods for developing mental health symptoms and disorders^[1,2]^. Athletes who were forced to retire because of injury may be twice as likely to develop common mental health symptoms than those who retired voluntarily.^[13]^ Retirement is a crucial and inevitable transition for all athletes, and career planning is a key aspect of athlete development. Support structures with longitudinal mental health surveillance and proactive management should be established during their active career, with post-retirement support, especially for those who are forced to retire prematurely. This is particularly important since many retired athletes of contact and collision sports report mental health symptoms^[14]^ and cognitive symptoms.^[15]^

## Mental health in the athlete’s entourage

Coaches, parents, friends, and the medical team play pivotal roles in an athlete’s mental health. Coaches help athletes to develop their physical and mental skills.^[16]^ They can detect poor mental health practices and should encourage the athlete to seek support for mental health symptoms.^[12]^ However, coaches may also experience mental health symptoms.^[16]^ They manage intense stress and navigate the pressures placed on them from the athletes, team, club, and management.^[16]^ The medical team can also experience mental health symptoms, which can be especially prominent at high-profile international competitions like the Olympic and Paralympic Games.^[17]^ These examples highlight the need for mental health surveillance and support in the broader athlete entourage, rather than only in the athletes.

## Fitting sports psychiatry into the broader mental health team

The diagnostic and management challenges of working with the mental health of athletes require an understanding of how elite athletes function, coupled with expertise and training in mental disorders. Examples of common complexities include challenges in differentiating primary depressive disorders from the overtraining syndrome or the persistent symptoms of concussion, as well as normal eating patterns from the spectrum of disordered eating and eating disorders that are common in athletes. Recently, changes in regulations will bring other complexities in athletes with disorders of sexual development (DSDs) and transgender athletes, where there are additional psychosocial stressors and potential iatrogenic psychiatric complications of endocrine treatments. These are circumstances where athlete-specific psychiatric care, treatment, and rehabilitation would be essential. Providing mental health care to South African athletes brings an additional layer of case complexity, requiring practitioners to embrace cross-cultural sensitivity and understand the individual in their context.

Sport and exercise psychiatry is a field that has been developing internationally for at least three decades.^[18]^ The field brings expertise in the diagnosis and treatment of mental health disorders in elite and recreational athletes, managing the performance aspects of mental health symptoms and disorders, as well as the use of exercise as a treatment for mental disorders in the general population. With any developing speciality, there is a political landscape to navigate as various new and established practitioners try to define their specific professional roles. However, like a team which is made up of many differently skilled individuals will often function best, managing mental health problems with such a multidisciplinary team is often highly beneficial. Psychiatrists with particular interest, expertise, and supplemental training in dealing with the intricate needs of athletes will be able to provide a key role in the diagnosis and management of mental disorders in this setting. With a medical background and mental health specialisation, psychiatry can often bring a holistic biopsychosocial view to inherently complex human issues.

Many individuals and sports organisations are looking for ways to access sport and exercise psychiatrists. We have called for the establishment of a Special Interest Group in the national body of psychiatrists, the South African Society of Psychiatrists, with the goal of formalising a network of practitioners. Task-sharing between specialists and generalists may allow for the most appropriate use of scarce expertise. Sports physicians may be well positioned to first assess and initiate treatment of mental disorders. Registered clinical psychologists, an equally scarce resource, would provide psychological treatment and work together with psychiatrists to triage care-level decisions for athletes with mental health disorders. Careful consideration should be applied in selecting the experts of the mental health team, due to the inherent vulnerability of work within the mental health space. As the field grows and develops further, delineating the boundaries in the scope of practice of the various mental health professionals and non-professionals will help organisations, athletes, and coaches access evidence-based services.

The modern era of elite sport requires an occupational health approach to managing its employees. The professional sports era has now been confronted with accusations of not sufficiently protecting its workforce from the risks of elite sports participation. These risks have been highlighted by recent cases of systematic athlete abuse by health practitioners and coaches, as well as the growing number of reported cases of traumatic encephalopathy syndrome in American football, rugby, and soccer. Organisations should bring mental health management firmly into view and include sport and exercise psychiatrists in the athlete entourage. Embedding the sports psychiatrist within the team and organisation, much like in the case of sports psychologists, tends to break down some of the initial barriers to trust and may facilitate mental health conversations. Dual loyalty with sports organisations is a challenge that needs to be actively managed. The athlete patient is at the centre, remaining the primary focus of the mental health practitioner. Confidentiality must be assured to allow for building a strong therapeutic alliance.

## Conclusion and recommendations

Athletes need experts who can manage the injuries that no one else sees. The stressors that athletes face are intense, with unique and complex circumstances. Beyond managing performance concerns, we need to have difficult conversations with athletes about mental health and life outside of sport, long before that final whistle blows on their sports careers. Mental health disorders are common in athletes and require specific attention and screening. While there are more athletes coming forward publicly about their mental health disorders, many still seem unable to access help. Sport and exercise psychiatrists can provide the expert mental health care required for athletes in South Africa in order to help round out the athlete entourage, with their complementary skills that bridge the artificial separation between physical and mental health. We provide seven recommendations on the way forward for sport and exercise psychiatry in South Africa:

1. Inclusion of sport and exercise psychiatry in the stakeholder meetings of sports organisations to advise on mental health policy and structures
2. Inclusion of sport and exercise psychiatry in the extended medical entourage for South African elite and non-elite athletes
3. Development of a referral network of sport and exercise psychiatrists in South Africa with clear referral channels
4. Training and upskilling of members of the athlete entourage to improve mental health literacy and allow for task-sharing
5. Further research into how mental health symptoms and disorders present and are managed in South African athletes
6. Further research into locally appropriate and acceptable mental health surveillance programmes that can be implemented into South African sports organisations
7. Establishment of local evidence-based guidelines for exercise as medicine for mental health disorders in the general population

Watch this space… because sport and exercise psychiatry’s South African journey has just kicked off.
